# Sperm protein profiles and their correlation with DNA integrity and protamine deficiency in Donggala bulls *(Bos indicus*): Implications for fertility assessment

**DOI:** 10.14202/vetworld.2025.2357-2366

**Published:** 2025-08-18

**Authors:** Abdullah Baharun, Hikmayani Iskandar, Tulus Maulana, Annisa Rahmi, Ristika Handarini, Ikhsan Qodri Pramartaa, Fitra Aji Pamungkas, Daud Samsudewa, Ekayanti Mulyawati Kaiin, Paskah Partogi Agung, Muhammad Gunawan, Yulius Duma, Raden Iis Arifiantini, Syahruddin Said

**Affiliations:** 1 Department of Animal Science, Faculty of Agriculture, Universitas Djuanda, Jl. Tol Ciawi, Bogor, West Java 16720, Indonesia; 2 Research Center for Applied Zoology, National Research and Innovation Agency (BRIN), Jl. Raya Bogor KM. 46, Cibinong, West Java 16911, Indonesia; 3 Research Center for Animal Husbandry, National Research and Innovation Agency (BRIN), Jl. Raya Bogor KM. 46, Cibinong, West Java 16911, Indonesia; 4 Department of Animal Science, Faculty of Animal and Agricultural Sciences, Universitas Diponegoro, Jl. Prof. Soedarto, Semarang, Central Java 50275, Indonesia; 5 Department of Animal Science, Faculty of Animal Science and Fisheries, Universitas Tadulako, Palu, Central Sulawesi 94119, Indonesia; 6 Division of Reproduction and Obstetrics, School of Veterinary Medicine, Biomedical Sciences, IPB University, Bogor, West Jawa 16680, Indonesia

**Keywords:** DNA integrity, Donggala bulls, fertility biomarkers, protamine deficiency, sperm proteins

## Abstract

**Background and Aim:**

The reproductive efficiency of livestock, especially indigenous breeds such as Donggala bulls, is pivotal to successful breeding programs. While conventional semen parameters are widely used, molecular markers, such as sperm protein profiles and DNA integrity, are emerging as reliable indicators of fertility. This study aimed to characterize the sperm protein profiles of Donggala bulls and examine their correlation with sperm DNA integrity and protamine deficiency.

**Materials and Methods:**

Frozen semen samples were collected from six Donggala bulls (aged 5–7 years). Pre-freezing evaluations included progressive motility (via computer-assisted sperm analysis), sperm morphology (using Diff-Quik staining), DNA integrity (assessed by the acridine orange assay), and protamine deficiency (assessed by the chromomycin A3 assay). Protein concentrations were determined using the bicinchoninic acid assay, and protein profiling was performed using 1D sodium dodecyl sulfate polyacrylamide gel electrophoresis. Band intensities and distributions were analyzed using ImageJ. Statistical correlations were analyzed using a one-way analysis of variance and Pearson’s correlation coefficients.

**Results:**

Significant individual variation was observed in semen quality among bulls. Progressive motility ranged from 38.3% to 46.1%, DNA integrity from 79.5% to 96.8%, and protamine deficiency from 96.0% to 98.7%. The number of protein bands per sample varied between 8 and 11, with molecular weights ranging from 5 to 175 kilodaltons (kDa). Protein concentration ranged from 8.32 to 20.70 μg/mL. A 35 kDa protein band was notably absent in one bull, which may be linked to lower motility. Strong correlations were observed between sperm motility and DNA fragmentation (r = 0.628), protamine deficiency (r = 0.539), protein concentration (r = 0.658), and protein band expression (r = 0.788).

**Conclusion:**

Sperm protein profiles in Donggala bulls are significantly correlated with DNA integrity and protamine deficiency, indicating their potential as molecular biomarkers for fertility prediction. These findings provide a foundation for integrating protein profiling into breeding soundness evaluations, suggesting that targeted proteomic analysis may enhance reproductive management strategies.

## INTRODUCTION

Donggala cattle (*Bos indicus*), an indigenous breed widely distributed in Central Sulawesi, Indonesia, are recognized for their strong reproductive performance [1]. Bull fertility, typically evaluated based on conception rates, plays a crucial role in the success of artificial insemination (AI) programs [2–4]. In Indonesia, frozen semen used in AI must meet national standards, including a minimum post-thaw progressive motility of ≥40% [5]. While conventional semen parameters such as motility, viability, membrane integrity, and morphological normalcy are commonly assessed, these indicators often fail to reliably predict male fertility outcomes[6].

Semen quality is significantly influenced by numerous proteins involved in spermatogenesis and sperm function[7]. Spermatozoa, essential for male reproduction and genetic inheritance, depend heavily on the integrity of their DNA and the presence of specific proteins that contribute to their fertilization capacity. During spermiogenesis, spermatids undergo essential cytogenetic transformations, including chromatin condensation and cytoplasmic reduction, which are critical for the formation of mature sperm [8, 9].

Protamine, a key nuclear protein, facilitates the tight packaging of DNA during chromatin condensation. A deficiency in protamine has been associated with abnormal chromatin structure and increased DNA fragmentation, resulting in impaired fertility [10]. Comparative studies across breeds such as Murrah, Silangit, and Toraya buffaloes have demonstrated that low protamine expression correlates with sexual infertility, highlighting its potential as a crossbreed biomarker for reproductive performance [10]. Further research should also explore additional nuclear parameters, including chromosomal abnormalities, DNA fragmentation, chromatin condensation defects, and nuclear vacuolation [11, 12].

Identification of specific sperm proteins and assessment of DNA damage have significantly advanced our understanding of sperm physiology and the molecular mechanisms underlying bull infertility [13]. The maturation process can increase reactive oxygen species (ROS) levels, contributing to DNA fragmentation [14]. In addition, seminal plasma proteins, which adhere to the sperm surface, modulate membrane structure and functionality and are potentially linked to male fertility [15, 16].

Spermatozoa contain a diverse array of proteins localized in the plasma membrane, flagellum, cytoplasm, acrosome, and nucleus, each contributing critically to various aspects of sperm function and male fertility [17]. These proteins have emerged as promising biomarkers for assessing semen quality. For instance, sperm protein profiling in Holstein bulls revealed five bands ranging from 55 kilodaltons (kDa) to 14.87 kDa, proposed as fertility-associated markers [18]. Similarly, Rosyada *et al*. [6] identified 15 sperm proteins in Madura bulls using liquid chromatography-tandem mass spectrometry.

Sperm proteins undergo topographic reorganization across surface regions, altering membrane properties through binding or molecular modifications [19]. Among these, protamines – mainly protamine 1 (P1) and protamine 2 (P2) – are central to chromatin condensation [8]. Their distribution varies across species: bulls, boars, rabbits, and domestic cats predominantly express P1 without P2, while some primate species express nearly 80% P2 [18]. Beyond chromatin packaging, these proteins are also involved in protecting sperm during fertilization [20].

Despite increasing recognition of the importance of sperm proteins in fertility assessment, limited studies have specifically focused on indigenous cattle breeds such as Donggala bulls. Most research to date has centered on widely studied commercial breeds, such as Holstein and Simmental, which differ genetically and physiologically from local breeds adapted to tropical environments. While semen quality parameters such as motility and morphology are routinely evaluated in Donggala bulls, molecular determinants – particularly sperm protein expression patterns and their functional correlations with DNA integrity and chromatin status – remain poorly characterized. Furthermore, the potential role of protamine deficiency as a predictive marker of fertility in Donggala bulls has not been systematically examined. These gaps hinder the implementation of precision breeding programs and the development of reliable molecular markers for reproductive soundness in indigenous livestock.

This study aimed to characterize the sperm protein profiles of Donggala bulls using one-dimensional sodium dodecyl sulfate polyacrylamide gel electrophoresis (SDS-PAGE) and evaluate their correlations with key semen quality indicators, includ-ing sperm DNA integrity and protamine deficiency. By integrating biochemical and cytological analyses, the study aims to identify potential molecular markers that may predict male fertility. The findings are expec-ted to provide foundational data for developing targeted reproductive management strategies and enhancing the genetic conservation of Donggala cattle through improved semen quality assessment protocols.

## MATERIALS AND METHODS

### Ethical approval

All procedures in this study were conducted in accordance with ethical standards and approved by the Animal Ethics Committee of the National Research and Innovation Agency (BRIN), under Approval No. 049/KE.02/SK/03/2023.

### Study period and location

This study was conducted from May to October 2024. Frozen semen was collected using an artificial vagina during mid-morning sessions following a routine weekly collection schedule at the Unit Pelayanan Teknis Daerah Pembibitan Ternak Sidera, Central Sulawesi Livestock Agency. Frozen semen samples were stored in containers filled with liquid nitrogen at –196°C and transported to the Genomic Laboratory of BRIN under cryogenic conditions for further analysis.

### Semen collection and inclusion criteria

Frozen semen samples were obtained from six Donggala bulls aged 5–7 years. Semen quality was evaluated based on volume, concentration, motility, and morphology. Only ejaculates meeting the minimum quality standards of ≥70% motility and ≥80% normal morphology were included in the study.

### Sperm motility and morphology assessment

#### Computer-assisted sperm analysis (CASA)

Sperm kinematics were analyzed using a CASA system. A 5 μL aliquot of semen was placed on a pre-warmed microscope slide (38°C), covered with an 18 mm × 18 mm coverslip, and examined under a 200× phase-contrast objective using an Axio Scope A1 microscope (Carl Zeiss, Germany) connected to Sperm Vision 3.7.8 software (Minitube, Germany). Images from eight microscopic fields were captured, and motility parameters were calculated from approximately 1000 spermatozoa/sample [21].

#### Morphological analysis

For morphological evaluation, 5–15 μL of semen was smeared onto a clean glass slide at a 45° angle and air-dried for ~15 min. The slide was then fixed by dipping it 5 times in Diff-Quik Fixative Solution (Solarbio, Beijing) and allowed to dry. Staining was performed by immersing the slide three times in Diff-Quik Solution I and five times in Solution II. After rinsing with sterile water and air-drying for ~30 min, a coverslip was mounted. Slides were observed under a 100× oil immersion objective microscope (Immersol, Carl Zeiss, Germany). Morphological abnormalities were classified according to Auger [22], including macrocephalus, microcephalus, pear-shaped heads, narrow heads, abnormal contours, abaxial insertion, detached heads, and bent midpieces or tails.

### Assessment of sperm DNA integrity and protamine deficiency

#### DNA integrity (Acridine orange staining)

Thawed semen was incubated at 37°C for 30 s, smeared onto glass slides, air-dried, and fixed in acetic alcohol (1 part glacial acetic acid: 3 parts methanol) for 2 h, followed by a second air-drying. Slides were stained overnight with acridine orange (diluted 1:1000 in phosphate-buffered saline (PBS) [23], rinsed with distilled water, sealed with synthetic resin, and examined under a fluorescence microscope (AxioVision, Carl Zeiss, Oberkochen, Germany) using a 490/530 nm filter. Spermatozoa with intact DNA were fluorescent green, while those with fragmented DNA exhibited yellow to red fluorescence.

#### Protamine deficiency (Chromomycin A3 [CMA3] staining)

To assess protamine deficiency, sperm were washed twice in PBS, fixed in Carnoy’s Fixative Solution (ethanol: chloroform:acetic acid, 6:3:1) for 8 min at 4°C, and air-dried. Slides were stained with 100 μL of CMA3 solution (0.25 mg/mL in McIlvaine buffer with 10 mM MgCl, pH 7.0) for 30 min at 4°C (Sigma-Aldrich, USA), then rinsed with McIlvaine buffer, dried, and mounted with antifade solution (Fluoprep, BioMerieux, France). Fluorescence microscopy (AxioVision; excitation 460–470 nm) was used to identify sperm with bright yellow fluorescence (protamine-deficient) and dull green/yellow fluorescence (normal protamine content) [23].

### Protein extraction and SDS-PAGE profiling

Thawed semen was washed three times in PBS (centrifugation at 1800 × *g* for 15 min) to remove seminal plasma. The resulting sperm pellet was lysed with PRO-PREP protein extraction buffer (iNtRON Biotechnology, Korea). Total protein concentration was determined using the bicinchoninic acid (BCA) method with the Pierce BCA Protein Assay Kit (Cat. No. 23225, Thermo Scientific, USA).

For profiling, proteins were separated using one-dimensional SDS-PAGE with 12% ExpressPlus precast gels (GenScript, Hong Kong) at 140 V and 75 mA for 55 min. Gels were stained with Coomassie Brilliant Blue, and molecular weights were determined using a Broad Multicolor Pre-Stained Protein Standard (M00624, ~5–270 kDa). Band intensity and expression were quantified using the ImageJ open-source software [24].

### Statistical analysis

All statistical analyses were performed using Minitab version 18.1 (Minitab Inc., USA). The Shapiro–Wilk test assessed normality, and Levene’s test evaluated homogeneity of variances. Data that met the assumptions for parametric testing were analyzed using a one-way analysis of variance, followed by Tukey’s *post hoc* test to determine significant differences among groups.

## RESULTS

### Sperm motility, DNA integrity, and protamine deficiency

Substantial variation in sperm quality was observed among the Donggala bulls, particularly in progressive motility, DNA integrity, and protamine deficiency. Progressive motility ranged from 38.3% to 46.1%, with the highest value observed in bull ID 105. DNA integrity ranged from 79.5% to 96.0%, with bull ID DK showed the highest percentage of intact DNA. Statistically significant differences in DNA integrity were detected among bulls (p < 0.05). Protamine deficiency ranged from 96.0% to 98.7%, with bull ID DB exhibited the highest deficiency (Table 1). These highlight significant individual variations in key semen quality parameters.

**Table 1:** Progressive motility, DNA integrity, and protamine deficiency (n=6).

Bull ID	Parameters (Mean ± SD)		

	Progressive motility (%)	DNA integrity (%)	Protamine integrity (%)
102	45.40 ± 5.10	92.40 ± 1.30^ab^	97.20 ± 2.47
103	44.70 ± 9.90	80.50 ± 6.40^c^	96.00 ± 0.71
104	38.30 ± 5.20	87.40 ± 6.30^bc^	98.20 ± 0.35
105	46.10 ± 9.80	92.80 ± 1.80^ab^	97.00 ± 2.12
DB	39.00 ± 7.40	79.50 ± 9.80^c^	98.70 ± 0.35
DK	44.30 ± 3.50	96.80 ± 1.20^a^	97.00 ± 1.41

Different letters within the same variable indicate a significant difference (p < 0.05). SD=Standard deviation

### Sperm morphological abnormalities

Significant differences in sperm morphology were observed among the bulls (p < 0.05). The most prevalent primary abnormality was the narrow head defect, which reached 14.75% ± 6.7% in bull ID 102. In contrast, the abaxial head defect was the least frequent (0.75% ± 1.5%) in bull ID 104 (Table 2). These findings highlight considerable morphological variability within the Donggala bull population.

**Table 2:** Sperm morphology of Donggala bulls.

Bull ID	Parameters (Mean% ± SD)								

	Pear shape	Narrow	Abnormal contour	Macrocephalus	Microcephalus	Abaxial	Detached head	Bent mid piece	Bent tail
102	4.75 ± 2.21^ab^	14.75 ± 6.7^a^	2.00 ± 1.15	6.50 ± 6.55^a^	3.25 ± 3.94	3.25 ± 0.95^ab^	7.25 ± 5.73	9.50 ± 1.91	9.75 ± 7.71^a^
103	1.25 ± 1.25^b^	9.75 ± 2.5^ab^	1.25 ± 0.50	1.50 ± 1.29^b^	7.25 ± 3.86	1.50 ± 2.38^b^	2.25 ± 1.25	8.25 ± 4.27	2.75 ± 3.09^b^
104	3.00 ± 2.58^ab^	12.00 ± 9.93^ab^	3.00 ± 2.44	4.25 ± 3.30^ab^	5.50 ± 6.40	0.75 ± 1.50^b^	8.25 ± 3.59	6.50 ± 3.69	2.00 ± 1.82^b^
105	5.50 ± 2.38^a^	4.75 ± 4.11^b^	2.25 ± 1.70	2.50 ± 3.10a^b^	2.25 ± 1.89	1.75 ± 1.25^b^	4.00 ± 4.08	5.50 ± 3.00	2.50 ± 2.08^b^
DB	1.25 ± 0.95^b^	4.25 ± 4.34^b^	2.75 ± 0.50	2.75 ± 0.95^ab^	2.25 ± 2.87	2.50 ± 1.29^ab^	4.00 ± 2.58	3.25 ± 1.50	0.25 ± 0.50^b^
DK	1.75 ± 0.95^ab^	6.00 ± 4.69^b^	2.50 ± 1.73	4.25 ± 0.95^ab^	2.00 ± 1.82	5.00 ± 2.70^a^	7.50 ± 5.80	10.00 ± 4.96	1.20 ± 51.25^b^

Different letters within the same variable indicate a significant difference (p < 0.05). SD=Standard deviation

### Frozen semen protein concentration

Protein concentrations in frozen semen varied considerably among individuals (Table 3). Bull ID 105 had the highest protein concentration (20.70 μg/mL), whereas bull ID 103 recorded the lowest (8.32 μg/mL). Additionally, the number of distinct protein bands varied between bulls, with bulls 102, DB, and DK each exhibited 11 bands, while bull 104 exhibited only 8 bands.

**Table 3:** Sperm protein concentration.

Bull ID	Frozen sperm protein	

	Concentration (µg/mL)	Bands
102	12.95	11
103	8.32	10
104	10.45	8
105	20.70	10
DB	13.71	11
DK	14.73	10

### Distribution of sperm protein bands

One-dimensional SDS-PAGE analysis of thawed semen samples revealed notable differences in protein band distribution among bulls (Table 4). Bulls 102 and DB expressed the greatest number of protein bands (11 bands), whereas bull 104 had the fewest (8 bands). Protein bands within the 13–30 kDa and 31–35 kDa molecular weight ranges were consistently observed across all bulls. Notably, a 35 kDa protein band was absent in bull 104 but present in all other bulls. Furthermore, a distinct 65 kDa band was detected in bull 105 but was not found in bulls 102 and 103, indicating inter-individual variation in protein expression profiles.

**Table 4:** Distribution of band proteins.

Molecular weight (kDa)	Bulls ID					

	102	103	104	105	DB	DK
5–14	+	+	+	+	+	+
15–30	+++	+++	++	++	++	++
31–35	++	++	++	++	++	++
35	+	+	-	+	+	+
36–50	+	+	+	+	+	+
51–64	+	+	+	+	+	+
65	-	-	-	+	+	+
66–95	++	+	+	+	+	+
130	-	-	-	-	-	-
175	-	-	-	-	+	-
270	-	-	-	-	-	-
∑ Bands	11	10	8	10	11	10

kDa=Kilodaltons, (-) indicates the absence of protein band expression), (+, ++, +++) represent the presence of one, two, or three of protein band expression).

### Correlations between protein expression and sperm quality parameters

Pearson correlation analysis demonstrated significant associations between sperm protein expression and key semen quality parameters (Table 5). Progressive motility was positively correlated with DNA integrity (r = 0.628), protamine deficiency (r = 0.539), protein concentration (r = 0.658), and protein band expression (r = 0.788). Additionally, protein band expression showed a strongly correlation with DNA integrity (r = 0.948) and a moderate correlation with protein concentration (r = 0.635). These findings suggest that specific sperm proteins may serve as potential molecular indicators of sperm integrity and functional competence.

**Table 5:** Correlation between sperm protein and sperm characteristic.

Parameters	Pmot	DNA integrity	Protamine deficiency	Protein concentration	Protein bands
Pmot	1	0.628*	0.539*	0.658*	0.788*
DNA integrity	0.628*	1	0.360	0.115	0.948*
Protamine deficiency	0.539*	0.360	1	0.337	0.264
Protein concentration	0.658*	0.115	0.337	1	0.635*
Protein bands	0.788*	0.948*	0.264	0.635*	1

* Significant correlation (p < 0.05), Pmot=Progressive motility

## DISCUSSION

### Overview and significance of the study

This study represents the first comprehensive evaluation of semen quality in Donggala bulls, integrating assessments of progressive motility, DNA integrity, protamine deficiency, and sperm morphology within the framework of breeding soundness examination. Most Donggala bulls met the Indonesian National Standard (SNI 4869-2:2021) for post-thaw motility (≥40%), except bulls 104 and DB, which had motility values of 38% and 39%, respectively. This suggests that these individuals have suboptimal sperm quality. Sulendre *et al*. [1] previously reported high sperm motility in Donggala bulls, correlating with improved fertility. With the exception of the two underperforming individuals, the semen quality of Donggala bulls exceeded that of Bali bulls [7], although it remained slightly lower than that of Madura [6] and Aceh bulls [25].

### Sperm maturation, energy demand, and genetic influence

Spermatozoa undergo maturation in the epididymis, during which they acquire progressive motility and fertilization capacity [26]. Spermatogenesis itself is a tightly regulated process that produces structurally and functionally competent sperm cells [26]. Given that sperm motility relies heavily on ATP production via mitochondrial activity, a high mitochondrial membrane potential is typically associated with enhanced fertility [27]. Genetic variation plays a substantial role in the phenotypic diversity of semen quality traits across breeds and individuals [28]. Interestingly, the repeatability of semen traits is generally higher than their narrow-sense heritability, indicating that environmental influences and permanent individual effects contribute more significantly to semen quality variation [29–32].

### Sperm DNA integrity and fertility implications

**Figure 1:**
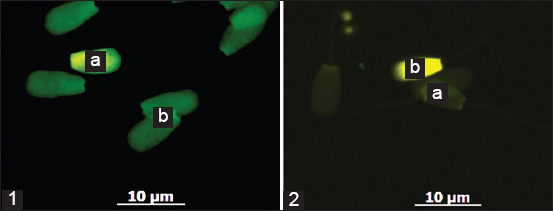
Magnification 60×, microscope fluorescence, Imager Z2, Carl Zeiss, Germany (1) DNA integrity, (a) Fragmented DNA (yellow-red fluoresence), (b) Intact DNA (green fluoresence) (2) Protamine Deficiency, (a) Normal/complete protamine (dark/dull green fluorescence), (b) Protamine deficiency (yellow fluorescence).

Sperm DNA integrity, a key indicator of semen quality [33], varied considerably among Donggala bulls ranging from 79.5% to 96.8% (Figure 1). This variation may be attributed to factors, such as oxidative stress, environmental exposures, and genetic differences. Reactive oxygen species (ROS), commonly produced by apoptotic spermatozoa, are known to be major contributors to DNA damage [34]. In dairy cattle, subfertile bulls have been reported to exhibit nearly twice the proportion of DNA-damaged sperm compared to fertile bulls [35]. Similar findings highlighting the significance of DNA integrity in relation to male fertility have also been reported in stallions [36, 37], boars [38–40], and rams [41, 42].

### Protamine deficiency and chromatin stability

Protamines, particularly P1 and P2, are essential nuclear proteins that facilitate chromatin condensation during spermatogenesis [43]. In the present study, Donggala bulls showed relatively high levels of protamine deficiency, ranging from 96.0% to 98.7%. These values surpass those reported in Bali bulls, in which normal and poor-quality fresh semen showed deficiencies of 93.45% and 95.64%, respectively [44]. Protamine deficiency impairs proper chromatin packaging and has been strongly associated with increased DNA integrity and reduced fertilization potential [8, 44].

### Sperm morphological abnormalities

Bull ID 102 exhibited a higher incidence of primary sperm abnormalities, particularly affecting the head and acrosome, structures typically associated with disrupted spermatogenesis [45]. According to Nagy *et al*. [46], sperm defects exceeding 18%–20% are considered detrimental to fertility; however, none of the Donggala bulls in this study exceeded this critical threshold. Ideally, sperm morphology observed under bright-field microscopy should display distinct structural regions, including the acrosome, midpiece, tail, and residual cytoplasm [47]. Deviations from normal morphology can impair sperm-oocyte interaction and compromise fertilization capacity [48]. Various environmental factors, such as temperature fluctuations and nutritional status, have been shown influence of morphological defects [49]. In particular, summer heat stress has been linked to an increased prevalence of abnormalities, such as knobbed acrosomes and nuclear vacuoles [50].

### Sperm protein concentration and band variation

**Figure 2:**
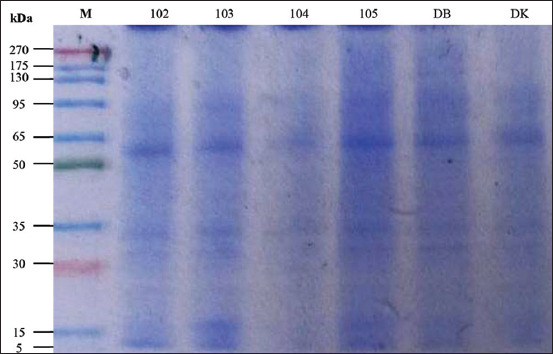
Sperm protein profile of Donggala bulls. kDa=Kilodaltons, M=Marker.

The average sperm protein concentration in Donggala bulls was lower than the values previously reported for Simmental bulls (35–56 mg/mL) [45]. Nevertheless, protein concentration alone is not a definitive indicator of semen quality [50], as protein function and subcellular localization are also critical determinants [51]. Notable variations in protein band intensity were observed among individual bulls (Figure 2), with thicker bands indicating higher protein abundance [52].

### Sperm quality traits and correlation with fertility

Key semen traits, such as sperm head and tail morphology, motility, and viability, exhibit a moderate correlation (~0.23) with insemination success [31, 53, 54]. Morphological abnormalities, including the presence of proximal droplets or structural deformities, may arise from impaired epididymal function or disrupted spermiogenesis [7, 38]. Additionally, semen processing techniques and the composition of extenders can further influence the expression of these defects. Notably, the heritability of sperm morphology traits, while moderate, is generally higher than that of female fertility traits [28].

### Protein band distribution and functional relevance

In this study, sperm protein bands ranged from 5 kDa to 175 kDa, with bull ID 104 notably lacking the 35 kDa band. This band corresponds to spermatid-specific manchette-related protein 1, a protein critical for spermatid elongation and the proper formation of the sperm head, acrosome, and tail [55, 56]. The absence of this protein may indicate structural or developmental defect that compromises sperm function. Protein-based biomarkers are useful for identifying elite sires and predicting progeny performance [51]. However, to full elucidate their biological significance, comprehensive proteomic profiling and functional characterization of sperm proteins are essential, particularly in relation to fertility, sperm physiology, and early embryo development [57–59]. Table 6 presents a list of candidate proteins identified from Donggala bull spermatozoa, with molecular weights ranging from 13.6 to 176.5 kDa. These proteins are implicated in key processes related to sperm function and fertility. Several proteins are directly associated with chromatin packaging and DNA integrity, such as histone H2B subacrosomal variant and Protamine-2 (PRM2), while others, including GPX4, contribute to antioxidant defense during spermatogenesis. Proteins such as IZUMO4 and SPACA1 are involved in sperm-oocyte fusion and acrosome function, essential for fertilization. Additionally, structural and motility-related proteins such as TUBA3C and HSP70 play roles in cytoskeletal organization and stress response. Other proteins, including AKAP3 and TMEM259, are associated with sperm maturation and signal transduction. The diversity of these proteins underscores the complex molecular mechanisms that govern sperm development, function, and fertility potential in Donggala bulls.

**Table 6:** Candidate sperm proteins of Donggala bulls.

Molecular weight (kDa)	Protein name
14.2	H2B subacrosomal variant histone
13.6	Protamine-2 (PRM2)
22.2	Phospholipid hydroperoxide glutathione peroxidase (GPX4)
24.3	Spermatogenesis associated with 3 (SPATA 3)
24.4	IZUMO family member 4 (IZUMO4)
32.8	Sperm acrosome membrane-associated protein 1 (SPACA1)
33	Proteasome assembly chaperone 1 (PSMG1)
35	Spermatid-specific manchette-related protein 1 (SMRP1)
35.3	Spermatid maturation protein 1
40.6 50.2	Recombinant binding protein suppressor of hairless Four and half LIM domains protein 1 (FHL1)
50.7	TUBA3C: Tubulin alpha chain
56.2	Interferon-inducible GTPase 5 (IRGC)
64.4	Spermatogenesis-associated protein 6
68.2	KRT2 A0A4W2GI13
70	Transmembrane protein 259
94.7	Heat shock protein 70 (HSP70)
176.5	A-kinase anchor protein 3 (AKAP3)
	Forkhead-associated phosphopeptide-binding domain

kDa=Kilodaltons

### Protein–DNA integrity associations and limitations

Previous studies in bulls [8], humans [60–63], and stallions [63] have demonstrated an association between specific molecular weight proteins and sperm DNA integrity. In particular, Fortes *et al*. [8] reported a strong association between reduced protamine content and increased DNA damage. Consistent with these findings, our study revealed a significant correlation between certain sperm protein bands and DNA integrity in Donggala bulls, reinforcing the potential of sperm proteomics as a diagnostic tool. Additionally, morphological abnormalities, particularly head defects, have been linked to both DNA fragmentation and chromatin deproteination [64]. Notably, sperm DNA fragmentation is increasingly recognized as an independent and complementary parameter in the comprehensive evaluation of semen quality [65].

### Limitations and future directions

However, the study was limited by a small sample size, the lack of protein identification through mass spectrometry, the absence of functional validation of identified proteins, and the lack of fertility outcome data, such as conception rates.

Future studies should include the use of mass spectrometry for definitive protein identification, functional assays to validate the roles of candidate proteins, integration of transcriptomic and proteomic analyses, and fertility trials to assess the predictive value of identified molecular markers. Expanding the sample size and population diversity would also enhance the reliability and applicability of the findings. Overall, this study advances our understanding of the molecular determinants of fertility in Donggala bulls. By establishing the correlation between sperm protein profiles, DNA integrity, and protamine deficiency, this study paves the way for the development of targeted biomarker-based tools for fertility prediction and the selection of elite sires in breeding programs. Such molecular insights are crucial for maintaining and enhancing the reproductive performance of local cattle breeds in tropical environments.

## CONCLUSION

This study is the first to comprehensively evaluate semen quality parameters, including progressive motility, sperm morphology, DNA integrity, protamine deficiency, and protein expression profiles, in Donggala bulls, an indigenous genetic resource of Indonesia. Sperm protein profiles in Donggala bulls are significantly correlated with DNA integrity and protamine deficiency, indicating their potential as molecular biomarkers for fertility prediction. These findings provide a foundation for integrating protein profiling into breeding soundness evaluations, suggesting that targeted proteomic analysis may enhance reproductive management strategies. These findings have direct application in AI programs and genetic improvement strategies, allowing for more informed selection of breeding bulls based on molecular and functional semen traits. Among the strengths of the study are its pioneering nature, the use of validated molecular assays, and the identification of protein bands potentially linked to fertility markers, such as the absence of the 35 kDa band in a subfertile bull.

## AUTHORS’ CONTRIBUTIONS

AB, HI, TM, and AR: Designed the study and drafted the manuscript. FAP, DS, RIA, EMK, and SS: Conducted the study, collected literature, and analyzed data. PPA, MG, and YD: Supervised field sampling and data collection. RH and IQP: Literature collection and data interpretation. All authors have read and approved the final version of the manuscript.
